# Design Optimization of a Submersible Chemiluminescent Sensor (DISCO) for Improved Quantification of Reactive Oxygen Species (ROS) in Surface Waters

**DOI:** 10.3390/s22176683

**Published:** 2022-09-03

**Authors:** Kalina C. Grabb, William A. Pardis, Jason Kapit, Scott D. Wankel, Eric B. Hayden, Colleen M. Hansel

**Affiliations:** 1 MIT-WHOI Joint Program in Oceanography, Cambridge, MA 02139, USA; 2 Woods Hole Oceanographic Institution, Marine Chemistry and Geochemistry, Woods Hole, MA 02543, USA; 3 Woods Hole Oceanographic Institution, Applied Ocean Physics and Engineering, Woods Hole, MA 02543, USA

**Keywords:** reactive oxygen species, superoxide, chemiluminescent, in situ analysis, ocean sensor, corals

## Abstract

Reactive oxygen species (ROS) are key drivers of biogeochemical cycling while also exhibiting both positive and negative effects on marine ecosystem health. However, quantification of the ROS superoxide (O_2_^−^) within environmental systems is hindered by its short half-life. Recently, the development of the diver-operated submersible chemiluminescent sensor (DISCO), a submersible, handheld instrument, enabled in situ superoxide measurements in real time within shallow coral reef ecosystems. Here, we present a redesigned and improved instrument, DISCO II. Similar to the previous DISCO, DISCO II is a self-contained, submersible sensor, deployable to 30 m depth and capable of measuring reactive intermediate species in real time. DISCO II is smaller, lighter, lower cost, and more robust than its predecessor. Laboratory validation of DISCO II demonstrated an average limit of detection in natural seawater of 133.1 pM and a percent variance of 0.7%, with stable photo multiplier tube (PMT) counts, internal temperature, and flow rates. DISCO II can also be optimized for diverse environmental conditions by adjustment of the PMT supply voltage and integration time. Field tests showed no drift in the data with a percent variance of 3.0%. Wand tip adaptations allow for in situ calibrations and decay rates of superoxide using a chemical source of superoxide (SOTS-1). Overall, DISCO II is a versatile, user-friendly sensor that enables measurements in diverse environments, thereby improving our understanding of the cycling of reactive intermediates, such as ROS, across various marine ecosystems.

## 1. Introduction

Understanding the response of organisms to stress is crucial for predicting the tolerance and adaptive capabilities of critical ocean ecosystems to global environmental changes. Reactive oxygen species (ROS), including superoxide (O_2_^−^) and hydrogen peroxide (H_2_O_2_), are recognized as vital compounds produced both as a byproduct of stress as well as a means for preventing stress [1,2,3,4,5,6,7]. This dynamic role that ROS play in organism health is largely dependent on the concentration and location of production [8]. Traditionally, ROS have been viewed as molecules that cause stress in organisms; when produced intracellularly, elevated concentrations are commonly linked to health decline and diseases within eukaryotes, possibly leading to cell apoptosis [1,2]. For example, the buildup of intracellular (IC) ROS is often postulated as a trigger of coral bleaching [9,10]. However, more recent research has illustrated that IC-ROS production did not lead to symbiont expulsion [11] and elevated temperatures did not necessarily produce elevated levels of IC-ROS [12,13,14,15], raising doubt about such a link between IC-ROS and bleaching. Additional research also highlighted the important health benefits of ROS; at lower concentrations, ROS is beneficial in physiological processes, such as micronutrient acquisition, cell signaling, and growth stimulation [3,4,5,6,7]. For example, when produced extracellularly at high concentrations, ROS can play a major role in the immune response to stress in processes, such as pathogen defense, antimicrobial effects, and physiochemical defense against heat stress, osmotic stress, and wound repair [16,17,18,19,20,21,22,23,24,25,26,27,28,29,30,31].

Due to the challenges presented when measuring superoxide, most of these health benefits associated with marine organisms have been observed in cultures and in laboratories [22,26,27,32,33,34,35,36]. As a radical species with a half-life in the order of tens of seconds, superoxide represents a particularly challenging molecule to measure in the field, limiting widespread characterization of its environmental concentrations and hindering studies of its distribution and behavior under natural conditions [34,37,38,39]. The pKa of superoxide is 4.88 and, therefore, at near-neutral pH, such as that within the ocean, the superoxide anion is the dominant form (O_2_^−^). Since superoxide does not pass through intact cell membranes [40], superoxide measured outside of organisms must be produced extracellularly [26,41]. A few studies have attempted to better understand superoxide production within natural environments by measuring extracellular (EC) superoxide within laboratory incubations [42], by extrapolating in situ production rates based on decay kinetics [43], and by using a benchtop instrument in shallow waters to make real-time measurements [41]. Despite such progress, these previous approaches fall short of measuring EC-superoxide production directly and are cumbersome, impractical, and restricted to calm, shallow water environments.

To directly measure superoxide concentrations in situ, we recently developed the diver-operated submersible chemiluminescent sensor (DISCO) [44]. This initial version of DISCO (hereafter referred to as DISCO I) is based on the same chemiluminescent method as a commonly utilized benchtop instrument, the FeLume [45]. The components used in the design of DISCO I were selected and/or manufactured to create a compact, submersible, handheld instrument that could be operated by a diver underwater. Initial field application of DISCO I provided the first in situ measurements of EC superoxide associated with coral reefs using a deployable sensor [44].

Direct measurements of superoxide within marine ecosystems may provide critical insight into the controls on organismal health. For example, EC-superoxide measurements associated with corals by DISCO I confirmed that coral EC-superoxide levels are species specific across several geographic regions [41,44]. Consistently high EC-superoxide concentrations associated with healthy coral were observed, with exceptionally high concentrations associated with species (i.e., Porites species) known to be stress and disease resistant [46]. These initial findings may point to EC-superoxide production as a beneficial process to coral health, possibly aiding in pathogen defense [44]. DISCO I enabled the measurement of EC superoxide at greater depths and across a wider variety of hydrographic conditions than was previously possible [41,44]. 

The goal of this study was to redesign DISCO II, which we present here along with a series of laboratory and field tests to verify the precision and accuracy of the instrument. DISCO II measures superoxide with a similar detection limit, yet improved sensitivity, and is more user-friendly, adaptable, and robust than DISCO I. Using DISCO I as a foundation, DISCO II is composed of a more advanced user interface, an improved fluidics module that draws less power, minimizing baseline drift and leading to a longer battery life, and an operation software that is easily adaptable. Contained within a smaller, lighter submersible housing, these improvements collectively contribute to a more robust and cost-effective sensor. These updates allow DISCO II to be more easily adopted by the scientific community and utilized across various aquatic environments, which will ultimately improve our understanding of these elusive chemicals in the environment.

## 2. Materials and Methods

### 2.1. DISCO II Design

**Figure 1:**
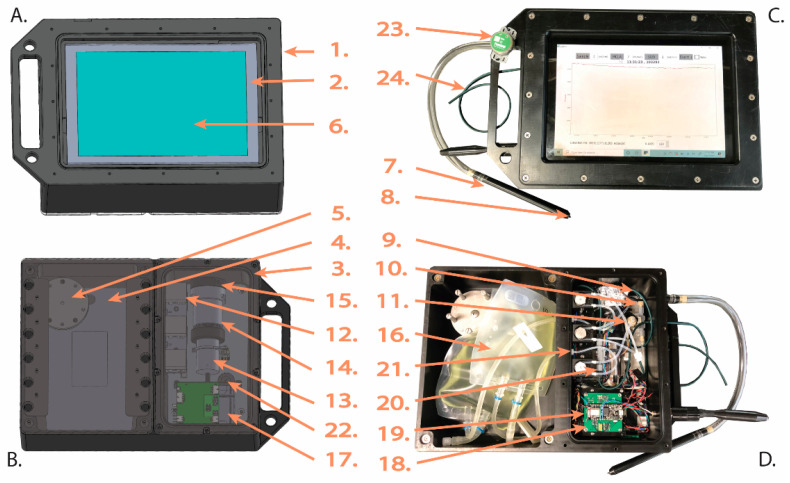
Diagram of DISCO II showing the (**A**) front and (**B**) back of the instrument alongside photos of the (**C**) front and (**D**) back. Numbers correspond with Table 1 and point to essential components in DISCO II.

DISCO II operates using the same principles as DISCO I [44] with important improvements to specific components and overall design (Figure 1 and Table 1). First, DISCO II contains three separate compartments: (1) a pressure-bearing compartment containing the electronics, fluid-handling pumps and lines, and photomultiplier tube (PMT), (2) an oil-compensated compartment containing a touch-screen tablet for user operation and data storage, and (3) a flooded compartment containing the reagent bags. Similar to DISCO I, DISCO II incorporates a sampling wand, which extends from the instrument to collect analyte waters, allowing the user to direct environmental samples into the instrument for analysis. The overall cost to reproduce DISCO is under USD 10,000, which is an improvement from the original DISCO I, which is estimated to cost over USD 15,000.

#### 2.1.1. Major Improvements from DISCO I

The major improvements from DISCO I primarily include replacing the large and power-hungry syringe pumps with low-power miniature peristaltic pumps and replacing the limited “push button” user control interface with a full-fledged diver-operated touchscreen tablet. The new smaller peristaltic pumps require much less space inside the pressure-bearing section. In addition, since they require less power, it is possible to power DISCO II using a small internal lithium-ion battery, instead of the larger external battery used in DISCO I. As a result, DISCO II measures 40.6 cm L × 27.9 cm W × 14.0 cm D and weighs 10.2 kg in air, or about 38% of the size and weight of DISCO I (both versions weigh around 1kg in seawater, salinity 35 g kg^−1^). 

The second major improvement in DISCO II is the touchscreen tablet user interface. In DISCO I, the instrument was controlled using a separate four-button handheld unit with a small display screen. In contrast, DISCO II incorporates a fully functional touchscreen tablet on the front of the instrument. This not only makes the instrument more compact but also provides vastly increased versatility and adaptability. DISCO II can be reconfigured for other software programs and the current user interface software can be amended and optimized for specific sampling parameters. The size of the screen also enhances the ability of the diver to view the signal and the touch interface facilitates a more straightforward and easier interaction.

A minor amendment to DISCO II includes the adaptation of the sample wand. DISCO II has a luer-lock attachment on the wand tip to enable sampling from a tubing connection, which facilitates in situ calibrations and decay rate measurements while increasing its versatility to sample from experimental set ups in lab-based experiments, for example.

#### 2.1.2. DISCO II Function and Layout

**Figure 2:**
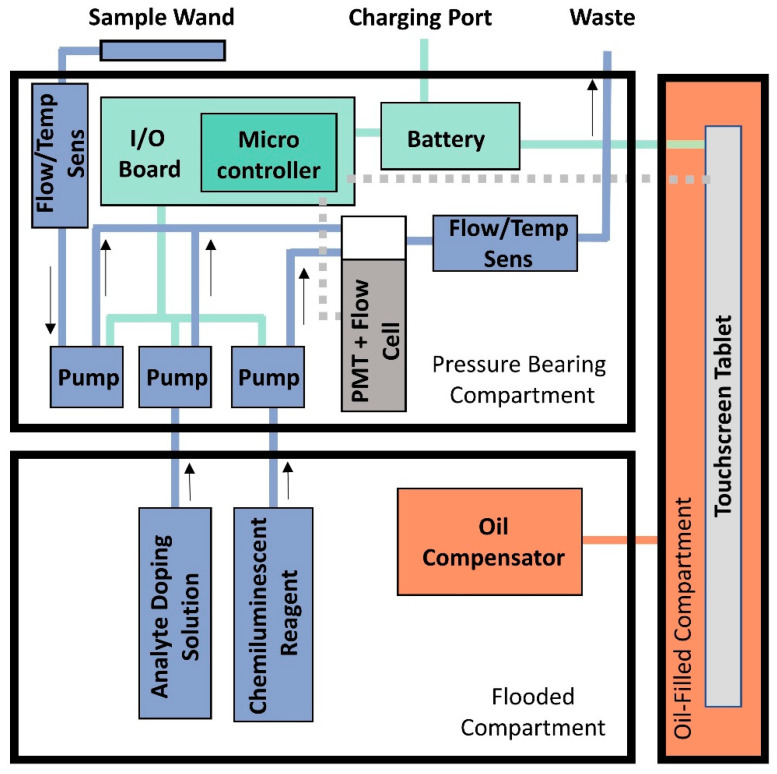
Block diagram of DISCO II showing the layout of DISCO and highlighting the function of the key features. The different operating systems are color coded, showing distinction between the fluidics system (blue), electronics system (green), the oil-compensated section (orange), and the tablet and PMT (gray). The other two compartments (pressure-bearing and flooded) are labeled accordingly. The blue arrows associated with the fluidics system indicate the direction of flow.

The overall function and layout specifics of the instrument are provided in the DISCO II layout (Figure 1) and block diagram (Figure 2). The flooded compartment contains two reagent bags (BioProcesss Container, Thermo Scientific Labtainer, Waltham, MA USA), one for the chemiluminescent reagent and one for a solution that can mix directly with the analyte fluid (analyte-doping solution). These reagent bags can be exchanged in situ while DISCO is in use, therefore, allowing for extended reagent use and/or multiple analyte-doping solutions to be used during the same sampling session. These two reagent bags have individual fluid lines that enter the pressure-bearing section via a bulkhead. Within the pressure-bearing compartment, the analyte-doping solution line merges with the analyte line, allowing the analyte-doping solution to be directly introduced to the sample stream. Depending on the analyte-doping fluid (i.e., SOD or SOTS-1), this can help confirm specific measurements (i.e., SOD scavenges superoxide allowing for target confirmation) and/or amend the analyte for chemical analysis (i.e., SOTS-1 can be used as a standard addition for calibration or to measure superoxide decay rates within waters). This analyte line then joins the chemiluminescent reagent fluid lines in a Teflon flow cell (made in house) where chemiluminescent measurements are quantified by a photo multiplier tube (PMT, H9319, Hamamatsu Photonics, Hamamatsu City, Japan). The flow of the three fluid lines (chemiluminescent reagent, analyte-doping solution, and analyte) are individually controlled by miniature peristaltic pumps (model p625Instech Laboratories Inc., Plymouth Meeting, PA, USA) which are also housed within the pressure-bearing section. The analyte inflow and waste outflow lines are also monitored by flow and temperature sensors (SLF3S-0600F, Sensirion Inc., Stäfa, Switzerland) which are used to confirm both the flow rates of each pump and measure fluid temperature.

The pressure-bearing section also contains a custom-designed embedded controller (dimensions 5.7 cm L × 5.7 cm W) that multiplexes communication between the tablet computer, the PMT, and pumps. The tablet communicates with an Adafruit Feather M0 microprocessor (Adafruit Industries, New York City, NY, USA) incorporated into the embedded controller via a Bluetooth to transistor-transistor logic (TLL) Universal Asynchronous Receive and Transmit (UART) serial bridge connection. The M0 voltage controls the three pumps with a peripheral I2C quad channel digital-to-analog converter (PN MCP4728). The M0 also acquires the signal from the PMT over a TTL to RS-232 serial connection. Data from the PMT are collected at 2 Hz (two data points per second) and are displayed in real time on the touchscreen tablet.

The tablet itself is a Samsung Galaxy Book 2 (Samsung, Suwon-si, South Korea) touch tablet that is contained within the oil-compensated compartment. The specific features that enable the touchscreen to function underwater deserve special attention and description here. Through testing, we discovered that “Super AMOLED” touchscreen technology could operate well in oil, whereas the screen for a standard AMOLED touchscreen tablet rapidly deteriorated by turning black and eventually failing altogether. We speculate the key difference is that the Super AMOLED technology eliminates an air gap between the tablet’s outer glass surface and the touch-sensitive layer, which protects it from oil ingress. The oil-compensated compartment is covered by a 0.015″ sheet of clear polycarbonate, which retains the oil while enabling touchscreen capabilities.

The graphic user interface (GUI) was developed in the Python3 environment using a Samsung Galaxy Book II. The PyQt5 python package was used for development of the GUI’s physical user interfaces including buttons, numerical inputs, and text display. The Matplotlib python package was used on top of the PyQt5 GUI to plot/display real-time data from the sensor. In the background the GUI handles data storage and wireless serial over Bluetooth communication with the embedded electronics in the sensor for control and data acquisition. This interface allows control of the pumps and PMT, enabling the user to operate the pumps by touch and to view the PMT signal intensity, fluid flow rates, and fluid temperatures in real time. The PMT settings (i.e., supply voltage to the PMT—i.e., voltage, and integration time for each datapoint—i.e., IT) can also be adjusted in real time to allow for instrument optimization. Although flow rates can be adjusted by the user in real time, the user interface is also adapted to automatically adjust the sample line flow rate to compensate for the additional flow added from the analyte-doping fluid line. All data collected are stored within the tablet and can be exported via Bluetooth or wireless internet connection. Power is supplied via a rechargeable lithium-ion battery (ND205XA4 Inspired Energy, Newberry, FL, USA). The lithium-ion battery life is longer than that of the tablet (battery life ~12.5 h) and, therefore, does not limit the overall battery life of the instrument. DISCO II can make measurements to a maximum depth of 30 m, making it ideal for underwater handheld use.

### 2.2. Chemical Components

While DISCO II can be optimized to measure different chemical species depending on the chemiluminescent probe, here, DISCO II is first adapted to measure superoxide. The chemical principles for measuring superoxide are based upon DISCO I [44] and the benchtop instrument, the FeLume [45]. Briefly, DISCO II utilizes a chemiluminescent probe, Methyl Cyprindina Luciferin Analogue (MCLA, Santa Cruz Biotechnology, Dallas, TX, USA), which has a high specificity for superoxide. The MCLA (4 µmol L^−1^) reagent is prepared with a 50 mmol L^−1^ sodium acetate buffer, which contains 50 µmol L^−1^ diethylenetriaminepentaacetic acid (DTPA) and is adjusted to a pH of 6, as described by previous protocols [34,39]. MCLA stock solutions are kept frozen at −20 °C until mixed with the sodium acetate buffer, which is prepared daily and kept at temperatures similar to the target environment. Photons generated by the MCLA chemiluminescent reaction are measured by the PMT in counts [47], which are then converted to superoxide concentration with daily benchtop calibration curves (described in further detail below). These calibrations use analyte fluid as a matrix and are conducted at the same temperature and pH as the water analysis.

Two different analyte-doping solutions were tested independently during instrument verification. To confirm the measurement of superoxide, superoxide dismutase (SOD) can be added into the analyte line (2.5 U mL^−1^ final, after mixing with analyte) at the end of a sample measurement. SOD is an enzyme that rapidly degrades superoxide to hydrogen peroxide and molecular oxygen [48]. The concentrated SOD stock solution (20 U mL^−1^) within the reagent bag is made in Milli-Q water and kept frozen at −20 °C until used. Alternatively, a standard solution can be doped into the analyte line to provide in situ calibrations and decay rates. Here, we used superoxide thermal source (SOTS-1) as a standard solution, which was synthesized to produce superoxide slowly and continuously over a long period of time [49]. A 100 µM SOTS-1 solution was prepared in aged, filtered seawater (AFSW, 0.22 µm filtered seawater that is aged in the dark for >12 h after the addition of DTPA) 10 to 16 h prior to use and stored around in situ temperatures to achieve a steady-state concentration [50]. To prepare this solution, SOTS-1 was first dissolved in argon-purged dimethyl sulfoxide (DMSO; 100 µL per mg) prior to dilution in AFSW.

### 2.3. Laboratory Verification

#### 2.3.1. Flow Rates

The fluidic system within DISCO II is optimized to pump analyte fluid at precise flow rates, delivering the sample fast enough to prevent decay, yet slow enough to allow analysis within the flow cell. Flow rates were analyzed over four days to evaluate stability of the precision. Tests were performed with DISCO II using DIW and flow rates were optimized within this study for subsequent superoxide measurements. The flow rate can be easily adjusted for other measurements via the user interface.

#### 2.3.2. Instrument Stability and Precision

Stability of DISCO II was assessed by measuring aged, filtered seawater (AFSW) in the laboratory for ~30 min at a flow rate of 4 mL min^−1^ (default voltage ~1000 V, 500 ms integration time). AFSW was prepared by filtering (0.22 µm) seawater amended with 75 µM DTPA and allowing it to sit in the dark overnight (>12 h) to complex and remove metals that might react with superoxide and interfere with measurements. Precision was assessed through percent variance, which was calculated by dividing the standard deviation by the average AFSW counts. This test also allowed for assessment of drift, as well as the influence of temperature and flow rates. 

#### 2.3.3. Instrument Optimization

Precision and reproducibility were evaluated by repeating a total of 107 calibrations across 10 testing days, which allowed for enough replicates to provide statistical analysis across time. Calibrations were conducted over a range of voltages (default, 800, 1200) and integration times (IT, 100, 500, and 900 ms). The default voltage (recommended setting) is optimized for each PMT individually at the factory and for this PMT, was determined to be ~1000 volts. The range of PMT settings was chosen because it spanned the settings used in previous studies (DISCO I: V default, IT 900; FeLume: V 1200, IT 100) [43,44]. All tests were conducted using AFSW from Martha’s Vineyard Sound. Daily calibrations were conducted according to previous methods [44] and used to convert superoxide chemiluminescent counts to concentration (pM). Briefly, due to the short half-life of superoxide, primary standard solutions were created immediately prior to each calibration. The primary standard solution was prepared by dissolving potassium dioxide (KO_2_) into a basic solution (0.3 N NaOH, 50–100 µM DTPA, pH = 12.5). The concentration of the primary standard solution was quantified by measuring the difference in absorbance at 240 nm on the UV-spectrophotometer before and after an addition of SOD (4 U mL^−1^, final). A secondary stock solution was prepared by adding 2 µL of the primary stock solution to 10 mL AFSW. The concentration of the secondary superoxide standard ranged from 2.4 to 19.1 nM. The chemiluminescent counts of the secondary stock solution were quantified on DISCO II at a flow rate of 7 mL min^−1^ by first measuring a baseline of AFSW until a steady-state signal was obtained (typically 1–2 min), followed by measurement of the secondary stock solution. During the measurement of the secondary stock solution on DISCO II the primary stock solution was quantified on the UV-spectrophotometer (240 nm). After ~1 min, SOD was added through the analyte-doping fluid line at a flow rate of 1 mL min^−1^ (2.5 U mL^−1^ final concentration) to confirm measurement of superoxide. 

Using these calibrations, instrument performance specifications were calculated across a range of PMT settings (Table 2). The AFSW baseline (raw counts) was calculated from the steady-state signal obtained prior to superoxide standard addition. The decay of the superoxide standard exhibited a pseudo-first-order decay with a log-linear distribution, from which the decay rate constant (s^−1^) and half-life (min) were determined [43,51]. Using the modeled fit of the decay kinetics and concentration of the primary standard, a calibration factor (pM count^−1^) and instrument sensitivity (count pM^−1^) were derived. The calibration factor can be used to convert chemiluminescent counts to pM. Since AFSW was treated to remove particles and metals, it served as an appropriate medium to calculate the limit of detection (LOD), which was calculated as three-times the standard deviation of the AFSW baseline signal. The percent variance for these measurements was also calculated by dividing the standard deviation by the average AFSW baseline.

#### 2.3.4. Versatility for Application

To verify the use of SOTS-1 within the analyte-doping fluid line and investigate the concentration measurements of superoxide observed with different integration times, we also measured SOTS-1 across time at a default voltage and IT of 100, 500, and 900 ms. SOTS-1 was added to AFSW through the analyte-doping fluid line at a flow rate of 2 mL min^−1^. With the addition of SOTS-1, the sample flow rate was automatically adjusted from 7 mL min^−1^ to 5 mL min^−1^ to compensate for the fluid addition and maintain a constant fluid flow rate through the cell of a total (analyte, analyte doping, and reagent fluids combined) of 14 mL min^−1^. All values are reported as background seawater (BGSW)-normalized SOTS-1 concentration (pM), which reflects the SOTS-1 signal minus the background AFSW signal, multiplied by the calibration factor that was collected prior to measurements.

To further assess robustness, DISCO II was tested by conducting calibrations with new luer-lock wand sampling tips as well as under a range of spatial orientations.

### 2.4. Field Verification

DISCO II was tested for in situ functionality at depth and across temperatures, as well as for an extended deployment to determine long-term in situ stability. To test the depth capabilities, DISCO II was used on SCUBA down to 20 m depth in Woods Hole, MA, and pressure tested and rated to 30 m. The function of the pumps has been previously used within the deep sea and, therefore, is suitable for shallow-water methods. DISCO II also measured in situ superoxide at temperatures from 19 to 33 °C during dives in Woods Hole, MA, and in the U.S. Virgin Islands. To evaluate long-term stability, DISCO II was operated continuously for ~60 min close to a shallow reef (depth ~5 m) off the coast of St. John’s, U.S. Virgin Islands.

### 2.5. Statistical Analysis

Statistical analysis was performed in Excel. Significant trends in data (i.e., if two PMT settings are related) were determined by calculating the coefficient of determination (R^2^), which was calculated for the linear regression between two variables. Single-factor ANOVA was used to determine if two groups of data (i.e., if PMT settings altered PMT counts) were significantly different from one another. *p*-values are reported to indicate statistical significance, with a *p*-value > 0.05 indicating that the two groups are not statistically different.

## 3. Results and Discussion

In comparison to DISCO I, DISCO II improvements presented here highlight a more sensitive, stable, and adaptable instrument. Here, we report on DISCO II’s performance through laboratory and field verifications. Overall, DISCO II, in comparison to DISCO I, showcases an improved user interface, better sensitivity, greater stability over time, and increased adaptability to diverse sampling conditions. 

### 3.1. Laboratory Testing

#### 3.1.1. Flow Rates

The stability of flow rates was vital for reliably delivering reagent and analyte fluids and achieving high-precision measurements. Flow rates ranging from 1 to 8 mL min^−1^ were measured over four days (to confirm continued stability) with a precision of ± 0.1 mL min^−1^. Superoxide measurements were conducted with a flow rate of 7 mL min^−1^ for both reagent and analyte fluid delivery, allowing samples to be measured within 27 s after entering the sampling wand. The flow rate of the SOD fluid line was 1 mL min^−1^ and SOTS-1 was added at 2 mL min^−1^. This range of tested flow rates and the sample delivery time were within the bounds of previously tested flow rates on DISCO I and the benchtop instrument [26,34,41,44]. The half-life of superoxide varies with environmental conditions and has been reported to range between 9.3 and 346.6 s [37,38,39,41,52]. Therefore, even at the fastest decay rates, DISCO II can measure superoxide within three half-lives.

#### 3.1.2. Instrument Stability and Precision

**Figure 3:**
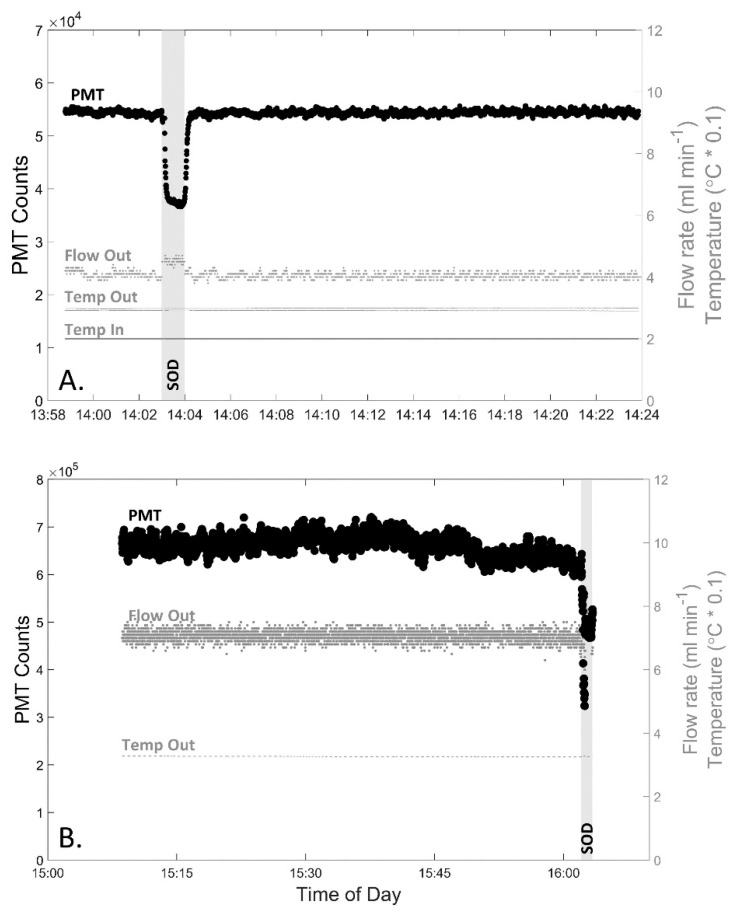
Raw DISCO II data during extended measurements to test for stability and drift within the instrument both in the (**A**) laboratory (~30 min) and (**B**) in situ near a shallow reef (~60 min), PMT settings are default voltage, IT 500ms. Graphs display the PMT counts (left y-axis, black large circles), effluent flow rate (ml min^−1^, right y-axis, grey small circles labeled “Flow Out”), effluent temperature (°C * 0.1, right y-axis, grey small dashes labeled “Temp Out”), and for graph A only, analyte temperature (°C * 0.1, right y-axis, grey small dashes for line on bottom labeled “Temp In”) throughout the time of day (x-axis). The time when the SOD pump is turned on is labeled and highlighted in a gray vertical bar.

Extended measurements of superoxide demonstrated that the instrument had an increased stability when compared to its predecessor, DISCO I. During the 30-min analysis of AFSW within the laboratory (Figure 3A), the percent variance of DISCO II was 0.7%, which was less than half the variance observed with DISCO I (1.90%) [44]. There was also no significant drift in PMT counts (R^2^ = 0.10), temperature (R^2^ = 0.737), or flow rate (R^2^ = 0.301). In comparison, pumps within DISCO I generated heat within the housing, leading to substantial baseline drift over time that required post-processing correction. The increased stability of DISCO II allowed for increased precision and accuracy over longer deployments and in situ measurements. Although the temperature trend across time was not statistically significant throughout this test, the temperature visually showed a slight change over time (−1.1 °C h^−1^). This minor temperature variation was not of concern, however, because each sample measurement was typically corrected to the baseline collected immediately prior, thereby accounting for any minor differences in temperature variation over the course of measurement collection. 

#### 3.1.3. Instrument Optimization

The 107 benchtop calibrations across 10 sample days demonstrated a similar detection limit and precision, with better sensitivity and improved reproducibility compared to the original DISCO I. While the calibrations were collected at a range of PMT configurations (voltage: default, 800, 1200; IT: 100, 500, 900 ms), the measurements that were independent of PMT settings (i.e., half-life, decay rate constant, LOD, and percent variance) were comparable to DISCO I (Table 2). Specifically, the average half-life of superoxide measured by DISCO II was 0.586 ± 0.215 min, which was comparable to that observed by DISCO I (0.28–1.10 min) [44]. Similarly, the decay rate constant (average 0.022 ± 0.008 s^−1^) and LOD (average 133.1 ± 87.4 pM) measured by DISCO II were within the range of specifications measured by DISCO I (0.01–0.041 s^−1^ and 111–116 pM, respectively) [44]. While it is noted that the percent variance of superoxide measurements by DISCO II during these calibrations (average 3.3 ± 1.0%) was higher than that observed above during the 30-min stability test (0.7%), this range of precision was still similar to DISCO I, which was 1.9% [44]. This variation in precision may be explained by minor undulations in signals, sometimes observed over short timescales, such as those at which the AFSW baseline is observed (~1–2 min). Although the sensitivity changed with PMT configurations, across all tested DISCO II settings, sensitivity ranged from 1.6 ± 0.8 to 104.9 ± 41.2 counts pM^−1^, typically orders of magnitude higher than DISCO I’s sensitivity (0.424 ± 0.116 counts pM^−1^). Across all calibrations, there were few outliers and calibrations were relatively consistent over varying sampling parameters, showcasing DISCO II’s ability to reliably reproduce measurements and perform similar to or generally better than DISCO I. 

**Figure 4:**
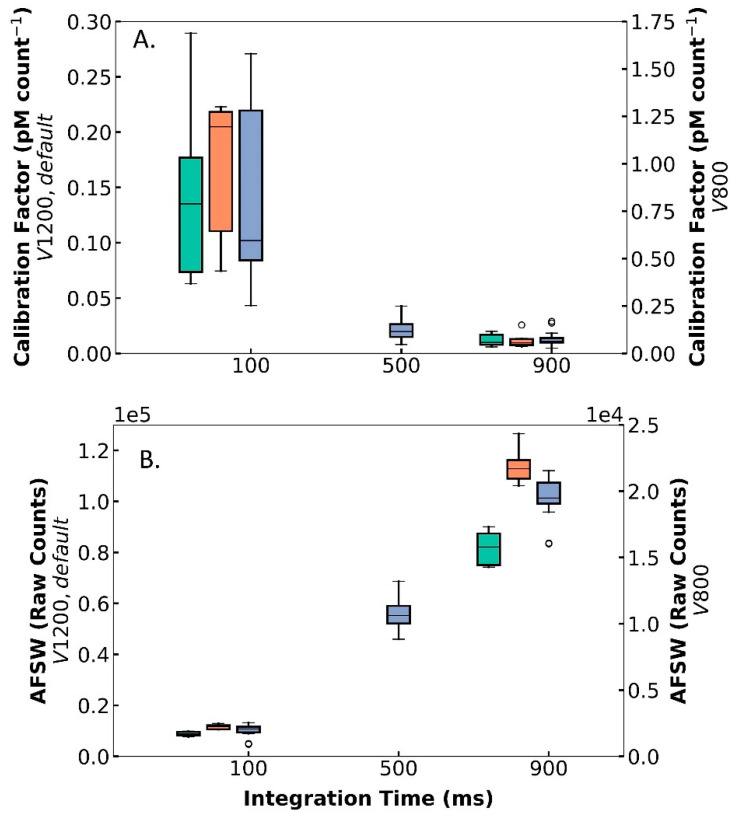
Results from multiple laboratory tests with DISCO, showing the (**A**) calibration factor (y-axis, pM count^−1^) and (**B**) AFSW raw counts (y-axis) across integration time (x-axis, ms) for different voltages (V): 800 (right y-axis, green), 1200 (left y-axis, orange), and default (left y-axis, blue). The box and whisker plot indicates the middle 50 percentile (box), the median (horizontal line), the middle 90 percentile (vertical lines), and the outliers (“o”).

While the PMT setting adjustments did not affect the precision and reproducibility of DISCO II, variations in the PMT supply voltage and integration time did alter the calibration factor, the sensitivity, and the baseline raw counts (ANOVA *p*-value < 0.05) (Table 2). Overall, the calibration factor (pM count^−1^) was inversely related to voltage and IT values, with lower voltage and lower IT yielding higher calibration factors (i.e., V 800, IT 100, calibration factor averaged 0.842 ± 0.463 pM count^−1^) and vice versa (i.e., V 1200, IT 900, calibration factor averaged 0.012 ± 0.007 pM count^−1^) (Figure 4A). Since sensitivity is inversely related to the calibration factor, it was expected that lower voltages and IT values would lead to lower sensitivity (i.e., V 800, IT 100, sensitivity 1.6 count pM^−1^) and vice versa (i.e., V 1200, IT 900, sensitivity averaged 104.9 count pM^−1^). Comparatively, the AFSW baseline signal was positively correlated with voltage and IT, with higher voltage and IT values exhibiting elevated AFSW counts (i.e., V 1200, IT 900, AFSW raw counts averaged 113,900 ± 6690 counts) and vice versa (i.e., V 800, IT 100, AFSW raw counts averaged 1695 ± 163 counts) (Figure 4B). For example, these data indicate that in environments with lower superoxide, DISCO II can be adjusted in real time to a higher voltage and IT to detect smaller changes in signals. Overall, the PMT settings within DISCO II can be optimized for specific situations without sacrificing the functionality, precision, and reproducibility of DISCO II.

#### 3.1.4. Instrument Versatility of Application

**Figure 5:**
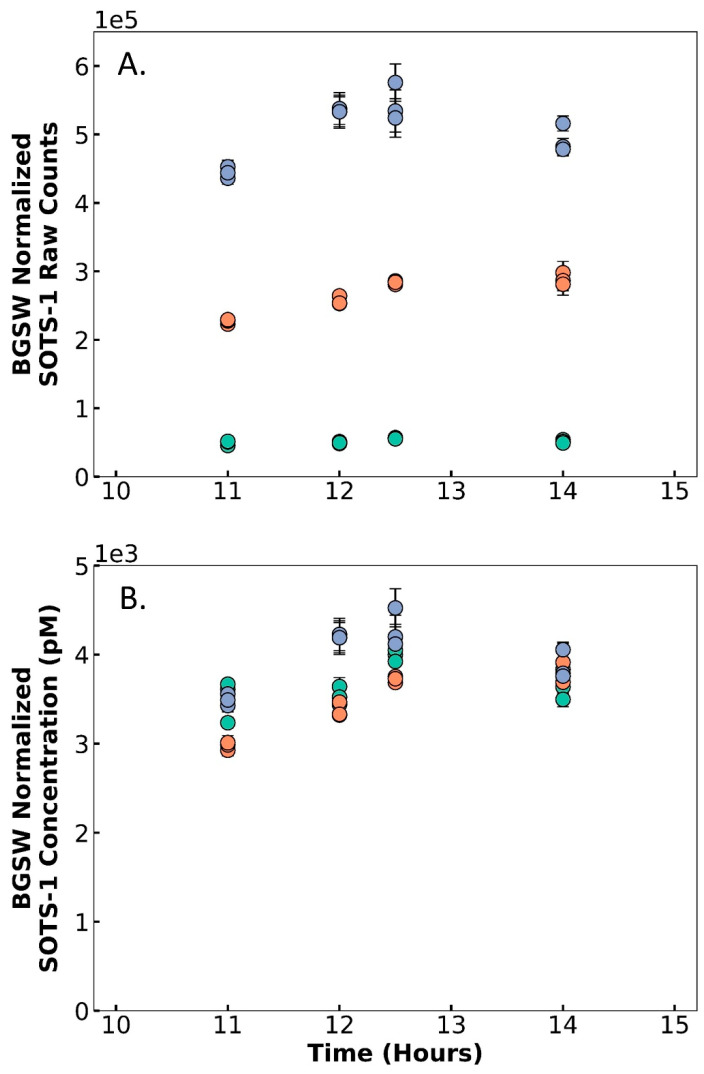
Results from multiple laboratory tests using SOTS-1 across time (x-axis, hours since SOTS-1 was mixed), showing (**A**) background seawater (BGSW)-normalized raw counts of SOTS-1 and (**B**) BGSW-normalized concentration of SOTS-1 (pM). Tests were completed using default voltage and different integration times: 100 ms (green), 500 ms (orange), and 900 ms (blue). Points represent the average signal and error bars represent the standard deviation.

Although the baseline raw counts change with PMT settings, the concentration measurements are not statistically different between integration times of 100 ms and 500 ms, demonstrating DISCO II’s ability to be used reliably across these different PMT settings (Figure 5). When measuring SOTS-1, the BGSW-normalized raw counts scaled proportionally with the integration time; an IT of 900 ms measured counts (average 476,700 ± 52,780 counts), nearly an order of magnitude higher than counts observed with an IT of 100ms (average 48,400 ± 6681), and those with an IT of 500 ms split the difference (246,400 ± 37,000). When applying IT-specific calibration factors (using KO_2_, as described above) that were determined directly prior to SOTS-1 measurements, the corrected concentration of SOTS-1 was similar across all tested integration times; average BGSW-normalized SOTS-1 concentration was 3723 ± 257 pM, 3489 ± 343 pM, and 3973 ± 315 pM, for IT 100, 500, and 900 ms, respectively. For IT values of 100 ms, no statistical differences were observed between measurements at IT values of 500 ms (ANOVA *p*-value = 0.085) or 900 ms (ANOVA *p*-value = 0.054). However, there was a statistical difference between IT 500 ms and 900 ms (ANOVA *p*-value = 0.002). Therefore, integration times of 100 ms and 500 ms can reliably be used interchangeably with comparable data as long as the calibration factors are completed using the same PMT settings. However, these tests indicate that longer integration times, such as 900 ms, might require further investigation to determine accuracy compared to lower integration times. Additionally, the use of SOTS-1 as a reliable superoxide concentration showcases SOTS-1 as a standard solution that can be used for in situ calibrations and decay rate measurements. Overall, these tests indicate that DISCO II can collect measurements at a range of PMT settings, allowing DISCO II to be versatile and adaptable to diverse coastal environments.

In addition to the internal settings, the adaptable luer-lock wand tip and flexible orientation make DISCO II versatile to collect measurements in a wide range of environments. The luer-lock wand tip addition did not impact superoxide AFSW raw counts (ANOVA *p*-value = 0.265) or calibration factors (ANOVA *p*-value = 0.772), therefore, allowing the wand tip to be interchanged without affecting measurements. Additionally, there were no significant differences in DISCO II superoxide measurements as a function of orientation (ANOVA *p*-value = 0.164 for AFSW raw counts, 0.073 for calibration factors), making it easy to use DISCO II in a variety of circumstances that might require different orientations (i.e., placing it upright on a lab bench vs. holding it at various angles between upright and horizonal while sampling within the environment).

### 3.2. Field Verification

DISCO II proved to function normally at depths up to 20 m and within the tested temperatures of 19 to 33 °C. Depths and temperatures outside of these ranges were not tested, yet we expect DISCO II would operate similarly within its 30-m depth rating and in colder environments. 

#### In Situ Stability Test

DISCO II was deployed for nearly 60 min in a near-reef environment to test the signal stability in seawater (Figure 3B). Overall, the BGSW was, on average, 663,000 ± 19,900 counts, equating to a percent variance of 3.0%. Throughout this time, there was no significant drift in PMT signal (R^2^ = 0.161). During these measurements, two distinct segments were noted that showed a decrease in counts towards the end. While it cannot be confirmed, it is likely that there were natural variations in superoxide within the ecosystem due to changes in photochemical production of superoxide as the sun lowered behind trees, creating a shadow during the ~60 min. Prior to these changes in signal, during the first ~30 min, the average BGSW was 670,900 ± 16,000 counts with a percent variance of 2.4% and there was still no significant drift in the PMT signal (R^2^ = 0.194). While these measurements take into account all real environmental changes, DISCO II exhibits about a third less variance than that of DISCO I in a similar near-reef environment (7.6%) [44]. During these measurements, the effluent from DISCO II recorded an average temperature of 32.6 ± 0.1 °C and a flow rate of 7.1 ± 0.2 mL min^−1^, confirming that the flow rate did not change significantly over time (R^2^ = 0.002). The temperature did show a slight decrease over time (32.8 to 32.5 °C over 60 min) (R^2^ = 0.819). While this temperature change is within the ~1 °C variation observed within laboratory testing, it may also be influenced by in situ temperature changes as well, since this test was completed between 1500 and 1600 local time.

### 3.3. Future Applications 

DISCO II could easily be adapted to make measurements on various platforms, using different fluid-intake approaches, and over more extended periods of time. In addition, pump operations could be pre-programmed to allow for deployment and autonomous operation of DISCO II within a system. For instance, such an operation modality would allow DISCO II to measure superoxide concentrations over the course of a diel cycle, providing insight on the role of light-dependent and light-independent superoxide production processes. The main limiting factor here would likely be reagent reactivity, which declines over a timeframe of several days; yet, this could be tested and characterized to adjust data accordingly. We also plan to use DISCO II as a foundation for developing a microfluidic-based chemiluminescent sensor to further decrease size and move towards deployable devices. 

Similar to DISCO I, DISCO II could also be adapted to measure other chemical species that have high-sensitivity chemiluminescent probes [44,53]. For example, DISCO II can be easily adapted to measure hydrogen peroxide by using the chemiluminescence of acrodinium esters (AE-CL method) [45]. The ability to measure both superoxide and hydrogen peroxide would allow for even more robust in situ characterization of ROS cycling. DISCO II could also be adapted for chemicals beyond ROS that can be measured via chemiluminescent probes, including species that are difficult to measure in situ, such as those with short half-lives (e.g., nitric oxide (NO), adenosine triphosphate (ATP), or that are redox sensitive (e.g., aqueous ferrous (Fe^2+^) and ferric (Fe^3+^) iron, and hydrogen sulfide (H_2_S)). The demonstrated versatility of DISCO II opens doors to understanding the distribution and quantification of elusive reactive chemicals.

## 4. Conclusions

This study highlights the improved operation, versatility, and adaptability of our previously designed handheld submersible chemiluminescent sensor (DISCO) [44]. Here, DISCO II was further adapted to enable the adjustment of PMT settings in real time, making measurements within diverse environmental settings possible. Improvements also allowed for in situ calibrations and decay rates. DISCO II reliably measured superoxide over several hours without drift or mechanical issues. The compact size and user-friendly operational interface make DISCO II accessible for all users across different types of coastal ecosystems. This ability to measure superoxide in situ across a wide variety of environments allows DISCO II to be integrated into a wide variety of field applications. These features are crucial for obtaining precise and real-time measurements of superoxide near production sources. Superoxide measurements of this type are necessary on a larger scale to truly understand the superoxide dynamics across marine environments and to characterize the role that superoxide plays in marine organismal health.

## Figures and Tables

**Table 1:** List of DISCO II components with description of the brand, model, details, function inside the instrument, and cost for all units. Numbers (Num) correspond with the components shown in Figure 1. The “*” indicates components that are updated from DISCO I to DISCO II.

Num	Name	Brand	Model	Details	Function	Cost for All Units
1 *	Dive housing	Made in-house	NA	Delrin housing, sealed water tight	Protects electronics and mechanical parts against water and pressure	USD 1500
2 *	Oil-Filled Tablet Compartment	Made in-house	NA	Delrin housing covered with clear 0.005″ thick PVC plastic front, flooded with oil	Houses tablet	Included in Dive Housing
3 *	Pressure bearing compartment	Made in-house	NA	Delrin housing with black plastic lid, sealed with O-ring and screws	Houses electronics, pumps, and PMT	Included in Dive Housing
4 *	Flooded compartment	Made in-house	NA	Delrin housing covered with black plastic lid with holes that allow water in, secured with magnets	Houses reagent bags	Included in Dive Housing
5 *	Oil compensator	Made in-house	NA	Silicone bellow attached to oil-filled compartment	Excess oil to compensate oil-filled compartment	USD 100
6 *	Tablet	Samsung	Galaxy Book 2	Hosts user interface and instrument control and communication, stores data	Protects electronics and mechanical parts against water and pressure	USD 400
7	Sampling wand	Made in-house	NA	Plastic tube with inlet tubing	Intake of analyte fluids	USD 150
8	Wand Filter Screen	Lee Fluidics	FSHF-2304025A	Filter screen with 0.010″ holes	Filter to prevent clogging and particulate matter from entering	Included in Wand
9	Tubing	McMaster Carr	Polyethelene Tubing	1/8″ OD × 1/16″ ID	Transports fluids from source through pumps, and flow cell	USD 20
10	Fangeless furrule fittings	IDEX Health and Science	P-235X	PEEK, 1/4-28 thread with flat bottom	Fit tubing to ports of dive housing, pumps, and flow cell	USD 100
11 *	Check valves	IDEX Health and Science	CV-3335	Peek, Chemically inert	Allows fluid to flow in one direction to prevent back-flow	USD 100
12 *	Mini peristaltic pumps	Instech Laboratories Inc.	p625	Three pumps with viton tubing	Three pumps for analyte fluid and chemical reagents	USD 2200
13	Photomultiplier tube (PMT)	Hamamatsu Photonics	H9319	2.54 cm diameter head-on PMT	Measures chemiluminescent signal	USD 1980
14 *	PMT Housing	Made in-house	NA	Covers sensing portion of PMT from stray light	Houses PMT	USD 700
15 *	Flow cell	Made in-house	NA	Spiral path, Teflon	Mixes analyte and reagent for PMT to measure	USD 100
16	Reagent Bags	Thermo Scientific Labtainer	BioProcess Container	plastic, 500 mL (MCLA) and 50 mL (SOD)	Holds chemical reagents	USD 30
17 *	Smart Lithum Ion Battery	Inspired Energy	ND205XA4	14.4 [V], 4 [A] maximum, 4.9 [Ah] capacity	Powers PMT, electronics, and tablet	USD 120
18 *	Micro Controller and I/O Board	Adafruit Feather	M0	Incorporated into custom built embedded controller (WHOI 3-0002-01), dimensions 5.72 × 5.72 cm	Communicates with tablet via Bluetooth. Controls voltage and acquires the signal from the PMT over an RS232 serial connection	USD 50
19 *	Battery Charger and Power Distributor	WHOI Acomms	204104, 205099	Power control board that control the battery charging and distribution of power to the microcontroller, PMT, and pumps	Power supply and management	USD 850
20 *	Flow and Temperature Sensor	Sensirion	SLF3S-0600F	Placed in-line and sends flow rate and temperature in real time to tablet via Bluetooth	Measures flow rate and temperature for analytical inlet and combined outlet	USD 250
21 *	Fluidics bulkhead	Industrial Specialties Mfg	PBHV-116-18-WN	Connects pressure-bearing and flooded compartments	Bulkhead to pass through reagent fluid lines	USD 200
22 *	Electrical bulkhead	CeramTec	Pressure: Ceramtight 50040-01-A	Connects pressure bearing and oil filled compartments	Bulkhead to pass through charging cord for tablet	USD 100
23	HOBO logger	Onset	MX2202	Temp/Light	Records temperature and light intensity	USD 100
24	Waste outlet	IDEX Health and Science	PEEK Tubing	0.159 cm OD, 0.076 cm ID	Discharges all fluids	USD 50
					Total Cost	USD 9100

**Table 2:** Summary of DISCO II calibrations within the laboratory at different voltages (800, 1200, and default) and integration times (IT: 100, 500, 900 ms). The average and standard deviation of results are displayed for calibrations conducted within aged, filtered seawater (AFSW). Displayed are the baseline counts prior to superoxide spike (raw counts), the calibration factor (pM count^−1^), the sensitivity (count pM^−1^), the half-life of superoxide during the calibration (min), the decay rate constant (s^−1^), the initial concentration of superoxide (pM), the limit of detection (pM), and the percent variance (%). The ANOVA *p*-value is indicated for each measurement across different PMT settings, with an “*” indicating statistical significance (*p*-value < 0.050). For those measurements that were not statistically different across PMT settings, the instrument (inst.) average and standard deviation across all settings are displayed.

PMT Setting	Voltage	800	800	1200	1200	Default	Default	Default	ANOVA *p*-Value	Inst. Average
	IT (ms)	100	900	100	900	100	500	900		
Count		6	6	5	6	31	27	26		
AFSW Baseline (Raw Counts)	Average	1695.4	15,715.7	11,568.9	113,858.7	10,261.5	56,483.1	101,393.2	* 5.47 × 10^−85^	
	Std. Dev.	163.7	1279.2	922.5	6687.2	2369.9	6176.4	7943.0		
Cal Factor (pM count^−1^)	Average	0.842	0.070	0.166	0.012	0.145	0.022	0.013	* 2.27 × 10^−25^	
	Std. Dev.	0.463	0.032	0.062	0.007	0.075	0.009	0.006		
Sensitivity (count pM^−1^)	Average	1.6	17.5	7.3	104.9	9.2	55.0	92.5	* 1.72 × 10^−23^	
	Std. Dev.	0.8	7.4	3.5	41.2	4.8	23.8	36.1		
Half-life (min)	Average	0.553	0.516	0.565	0.524	0.633	0.599	0.557	0.772	0.586
	Std. Dev.	0.330	0.244	0.155	0.178	0.258	0.147	0.177		0.215
Decay Rate Const (s^−1^)	Average	0.030	0.026	0.023	0.025	0.021	0.020	0.023	0.054	0.022
	Std. Dev.	0.016	0.009	0.008	0.008	0.008	0.004	0.007		0.008
Initial Superoxide Conc. (pM)	Average	10,748.5	9114.1	11,285.0	7831.7	8409.4	7785.9	7514.8	0.210	8307.3
	Std. Dev.	3262.3	4725.3	3161.1	2890.2	3243.5	3300.6	2558.3		3345.6
Limit of Detection (pM)	Average	163.2	96.1	168.7	149.5	144.9	132.5	110.7	0.545	133.1
	Std. Dev.	88.8	42.7	53.5	58.9	113.0	90.3	51.8		87.4
Percent Variance (%)	Average	3.9	3.0	3.0	3.9	3.2	3.5	2.9	0.110	3.3
	Std. Dev.	0.4	0.5	0.5	0.7	0.9	1.3	0.7		1.0

## Data Availability

Data is available within this paper.
